# Enhanced control structure design for an industrial off-gas system: Simple reconfigurations benefit the economy

**DOI:** 10.1016/j.heliyon.2023.e12934

**Published:** 2023-01-17

**Authors:** Feifan Shen, Lingjian Ye, Hongwei Guan, Yuchen He

**Affiliations:** a NingboTech University, Ningbo, 315100, China; b Huzhou Key Laboratory of Intelligent Sensing and Optimal Control for Industrial Systems, School of Engineering, Huzhou University, Huzhou, 313000, China; c Key Laboratory of Intelligent Manufacturing Quality Big Data Tracing and Analysis of Zhejiang Province, China Jiliang University, Hangzhou, 310018, China; d Ningbo University of Finance and Economics, Ningbo, 315175, China

**Keywords:** Off-gas process, Energy saving, Control structure design, Self-optimizing control, Controlled variable

## Abstract

For control structure design of the industrial off-gas benchmark system, application of the Skogestad's state-of-art design procedure has suggested the scrubber inlet pressure (*P*_si_ in the roaster) and one of the fan speeds (*N*_fan1_ or *N*__fan2_ in the furnace) as the self-optimizing controlled variables CVs. In this study, we stress and advocate the gSOC-plus-BAB approach as an enhanced design toolkit for the classical and systematical design procedure. The gSOC (global self-optimizing control) is able to efficiently solve measurement combinations as CVs with improved economic performances, while the BAB (branch and bound) algorithm serves to fast screen promising measurement subsets for large-scale problems. Using the enhanced design for the off-gas system, our new findings are to control the combination of roaster's ID fan outlet pressures, 0.494*P*_IDfan1_+0.506*P*_IDfan2_ (setpoint: −257.04 Pa), and the furnace's fan pressure difference, *P*_IDfan1_- *P*_IDfan2_ (setpoint: 0). Such simple reconfigurations can dramatically reduce the average economic loss by 53.3% for the roaster and even achieve perfect optimal control for the furnace. Both steady state and dynamic evaluations are carried out to validate the reconfigured control structures.

© 2017 Elsevier Inc. All rights reserved.

## 1 Introduction

With the increasing global competitions and stringent environmental regulations, integration of control and optimization of chemical and energy processes has been becoming vital in the modern industry [1,2]. The control systems play the core role for safe and efficient operations for chemical processes. Traditionally, the design of control systems often follow suggestions from chemical designers, who often determine controlled variables (CVs) and manipulated variables (MVs) based on their expert knowledge or additionally with mild quantitative analysis. Although relying on process empirical experiences can be easily understood and explained, the optimal decision is generally difficult to obtain. On the other hand, in the field of control engineering, researchers used to work intensively on controller design and parameter tuning, or try to develop advanced control strategies. However, in case that the control structure has not been properly configured in the first place, even the most sophisticated controllers or advanced control algorithms may fail to reach satisfactory control/optimization performance.

**Fig. 1:**
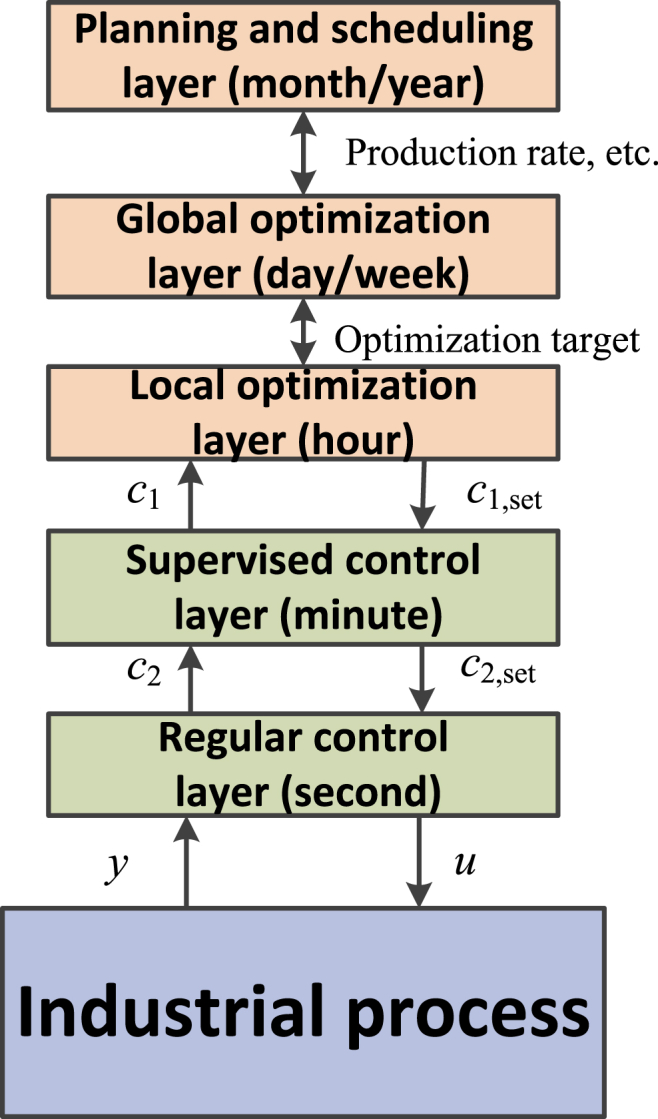
Hierarchical control structure for chemical processes.

In chemical industry, the hierarchical control structure has been widely adopted, which are cascaded by the control layer and optimization layer running at different time scales, as shown in Fig. 1. The control layer may further contain a regulatory control layer (second) and supervised control layer (minute), and the optimization layer is often composed of a local optimization layer (hour), a global optimization layer (day/week) as well as the planning and scheduling layer (month/year). These layers are linked and interacting via the CVs and their setpoints. Especially, the primary CVs, *c*_1_, which connect the control layer and the optimization layer, are of paramount importance for the entire control system. In the lower regulatory control layer, the secondary CVs, *c*_2_, are mainly for the stabilization purpose, such that local disturbances can be efficiently rejected.

The objective of control structure design (CSD) is to make “structural” decisions for configuring the architecture as shown in Fig. 1. Such decisions are especially critical for large-scale processes with massive operation units [[3], [4], [5]], which contain the following tasks [6,7]: 1) Selection of MVs *u*; 2) Selection of measured variables *y* (sensor positions); 3) Selection of CVs (including primary CVs *c*_1_ and secondary CVs *c*_2_); 4) Configuration of control loops; and 5) Selection of controller types (PID, model predictive control, etc.). Research on the CSD can be traced back to the 1960s for discussions on the process control techniques by Buckley [8]. Since then, CSD has attracted much attention from the community, and fruitful designing methods were proposed by researchers, see e.g. several comprehensive reviews [3,9,10].

Across the entire control structure shown in Fig. 1, the selection of CVs is crucial, especially the primary ones, *c*_1_. For the control layer, *c*_1_ are the ultimate CVs that are maintained at setpoints. While for optimization layer, *c*_1_ directly relates to the economic objective as their setpoints are the decision variables for economic optimizations. In general, *c*_1_ establishes the link between the process control and optimization. Conventionally, most CSD methods emphasized the system feasibility and stability, while they rely on the independent optimization layer to update the setpoint of *c*_1_ for optimality. In some sense, such methods have weakened the relationship between the control and optimization. In reality, however, the chemical processes are always affected by various uncertainties. Thereby, the predetermined setpoints are no longer optimal due to the change of operating conditions. Although constant re-optimization of the setpoints can restore the process optimality in principle, the optimization rate is often slow in the hierarchical control structure [11,12]. In this context, keeping CVs at some given setpoints should be reconsidered from an optimization perspective.

Among various CSD methodologies, the self-optimizing control (SOC) aims to select suitable primary CVs, such that the plant operation can be automatically optimized via regulatory control in spite of disturbances [7]. Generally, process variables whose optimal values are insensitive to disturbances are good CV candidates. Based on this principle, the Skogestad's state-of-art CSD procedure [13] was proposed, and it has achieved a tremendous success, both in academia and industry. Later, a number of successful applications using the design procedure were reported. However, this classical procedure still suffers from some suboptimality, owing to using single measurements as CVs. In order to reduce the suboptimality, the SOC methodology has been further advanced by using the measurement combinations as CVs [14,15]. In this context, this paper wishes to make a timely update for the approach. More specifically, we recommend the recent global SOC (gSOC) method [16], which turns out to be especially efficient and suitable for large-scale problems [[17], [18], [19]]. Compared with other SOC approaches that can also identify measurement combinations [11,14,[20], [21], [22], [23], [24], [25]], it has the following advantages: (1) The gSOC is not restricted to linear models and its self-optimizing performance is global; (2) Discrete operating conditions are handled independently (scenario-based), while continuous operating space can be sampled by the Monte Carlo method; (3) An analytical solution is available to solve the optimal combination matrix. For plantwide processes, the use of gSOC is further strengthened by the branch-and-bound (BAB) algorithm that can identify the best measurement subset, which is a complicated combinatorial problem. With these recent advances, it is therefore desired to enhance the classical CSD produce [13] by updating the CV selection step with the gSOC-plus-BAB method, such that the economic optimality could be enhanced for industrial applications.

In this paper, we will investigate the CSD problem for an industrial off-gas system, namely, the Xstrata Nickels Sudbury Smelter cleaning system described by Refs. [26,27]. The off-gas system is an important chemical sector in smelting industry for processing hazardous gas containing SO2 and CO. Due to the increasingly stringent environmental regulations, a fundamental objective is the control of leakage of harmful components into outside environment. Furthermore, the economic incentive to minimize the energy consumption is also urgent with the modernization of process integration. Previously, intensive researches have been placed on the process modelling and control of the furnace [[28], [29], [30], [31]]. These publications deal with problems prior to the installation of a control structure, or assume that the control configuration has been determined in the first place. On the contrary, limited works were devoted to the identification of an efficient control structure for the entire off-gas system, especially for economic optimizations. In Ref. [32], an optimization algorithm for the off-gas system was reported to find the operating strategy.

Pioneer works emphasizing on the CSD of the off-gas system were mainly carried out by de Araujo and his coworkers [27,33,34], who thoroughly followed the Skogestad's systematical procedure [13]. Both the roaster [33] and furnace [27] subsystems were investigated in-depth. In their studies, the self-optimizing CVs were finally identified as the scrubber inlet pressure (*P*_si_ in the roaster) and one of the fan speeds (*N*_fan1_ or *N*_fan2_ in the furnace), respectively, for the two subsystems. Both steady state calculations and dynamical simulations were carried out to show their effectiveness of the economic performance. These two reported configurations seem to be the most prominent control structures for the off-gas system in open publications up to date. Nonetheless, since the classical design approach was followed, these self-optimizing CVs are still single measurements, thus leaving some room for economic improvements. Motivated by the existing suboptimality, in this paper, the enhance CSD is applied to pursue better economic operations for the off-gas system. The focus is placed on the identification of measurement combinations as CVs to improve the economy, for both the roaster and furnace subsystems. Following the renewed CSD approach, it turns out that the derived configurations are sufficiently simple, and their performances are substantially better than the results in Refs. [27,33], validated by both steady state and dynamic evaluations. Hopefully, the proposed novel control configurations for the off-gas system could be used as promising alternatives by industrial practitioners in this field.

The rest of this paper is structured as follows. In Section 2, the Skogestad's systematical design procedure is briefly reviewed, with some discussions on its limitations. Section 3 presents the main modifications on the design procedure, then the gSOC method for identifying measurement combinations is introduced, together with a BAB algorithm for screening measurement subset. The results and discussions for reconfiguring the off-gas system are presented in Section 4. Finally, some conclusions are drawn in Section 5.

## 2 The systematical CSD procedure

### 2.1 Overview of the classical design procedure

The systematic CSD method by Skogestad [13] is based on the SOC principle. Currently, it is one of the most widely spread design methods, and has been successfully applied to a number of plant-wide chemical processes, for example [[35], [36], [37], [38], [39], [40]], to list a few. The procedure is briefly illustrated as follows [13]:

(1) Top-down analysis: selection of primary CVs:
  1.1 Define the operational target for the whole process;
  1.2 Analyze degrees of freedom;
  1.3 Select the primary CVs (elaborated in Section 2.2);
  1.4 Production rate setting;
(2) Bottom-up design:
  2.1 Design regulatory control layers which serves to, stabilize the process, reject local disturbances, select secondary CVs and pair the system inputs and outputs;
  2.2 design supervisory control layers: determine centralized or distributed control, etc.;
(3) Design optimization layers;
(4) Evaluate the performance.

The objective of step (1) is to improve the steady-state economic behavior via selection of appropriate CVs, and step (2) mainly concerns with the dynamic controllability/stability analysis. Compared with other more traditional CSD tools, this design procedure takes more advantage of process mathematical model instead of expert experiences, thus improving the universality of the method. The core idea inside the design procedure, namely, the SOC methodology, focuses on the plantwide process optimization via selecting the primary CVs. With the correct CVs, the regulatory control strategy is able to realize optimization while maintaining stabilization, hence answering the question of what variables to control from the perspective of optimization. Note that the traditional optimization strategies configure independent optimization layers to update the setpoints of CVs in Step (3), while it is unnecessary for the SOC to (frequently) do such updating. When the loss is sufficiently small, the installation of an optimization layer can even be skipped, thus substantially reducing the system complexity. Moreover, since the functional frequency of the control layer is much higher than the optimization layer, the SOC efficiently compensates the delay of traditional optimization strategies, leading to the overall optimization performance improved.

### 2.2 Selection of primary CVs (step 1.3)

This paper focuses on the step 1.3 within the described CSD procedure, namely, the selection of CVs (for simplicity, the CVs in the rest of this paper refer to the primary CVs, otherwise noted). The mathematical framework developed in Ref. [13] for step 1.3 is outlined as follows.

Suppose that the operational economic objective for a chemical plant is formulated as the following constrained optimization problem (Eq. (1)):

(1) $$\begin{array}{l} \min_{u}\;J\left(u,d\right)\\ s.t.\;g\left(u,d\right)\leq 0 \end{array}$$

where *J* is the economic index determined in step 1.1, *u* and *d* are MVs and disturbance variables, respectively, *g*(*u, d*) are the process constraints. Besides, the input-output model of the system is (Eq. (2))

(2) y=f(u,d)

where *y* are the measured variables, *f* represents the model function.

Assumption 1 The mathematical representations for process models, Eqs. (1) and (2), are known.

Assumption 2 The operating conditions that may be encountered in operations are *expectable*, represented by some disturbances variables, d. (Here, d could be either parametric or scenario-based.)

The CVs are selected as two types of variables, *c*_1_ = [*c*_a_ *c*]^T^, where c represent variables located at the constraint boundaries (active constraints) and c are those inside the feasible region. Commonly, *c*_a_ could be process variables related to the product quality and safety conditions, which are typically constrained at the boundaries. Such variables are also likely to be identified by the conventional heuristic CSD methods. On the contrary, the selection of c is much more implicit, which constitutes the main concern by the SOC.

Assumption 3 The active constraints, *c*_a_, keep invariant when the operating conditions change (for different values of d).

Under Assumption 3, it is allowed to firstly assign part (the same degrees of freedom) of the MVs to control *c*_a_ at their boundaries, then the problem can be converted to an unconstrained optimization problem in the reduced space (Eq. (3)) [41]:

(3) $$\min_{u}\;J\left(u,d\right)$$

where, for the sake of simplicity, the notation u is still used to represent the rest MVs.

The loss function L is defined as (Eq. (4)):

(4) $$L=J^{fb}\left(d\right)-J^{\text{opt}}\left(d\right)$$

where *J*^fb^ is the closed-loop economic performance and *J*^opt^ is the optimal cost for a given d. For a set of candidate CVs, c with setpoints of *c*_s_, *J*^fb^ is evaluated as (Eq. (5))

(5) $$\begin{array}{l} J^{fb}\left(d\right)=J\left(u,d\right)\\ s.t.\;y=f\left(u,d\right)\\ \;c=c_{s} \end{array}$$

for a given d. Then the following steps are followed in sequence to identify self-optimizing CVs [7,13]:

1.3.1 Define possible disturbance scenarios or operating conditions for the chemical process (Assumption 2);
1.3.2 Determine the candidate set of the CVs, *c*⊂{*y*};
1.3.3 For all possible c⊂{*y*}, calculate the total/average closed-loop loss L under all operating conditions, computed per (4) and (5);
1.3.4 Select several groups of CVs with minimum total/average loss for further evaluations, such as dynamic verifications.

This routine has been successfully applied on the CSD for many plant-wide chemical processes as introduced earlier. These researches demonstrated that the economy-oriented SOC is advantageous over the heuristic CSD methods in improving the economic performance.

### 2.3 Restrictions

The CVs selection method in the above state-of-art design method is however restricted by a few factors: (1) The CVs are generally individual physical variables (except for some cases, certain physical relationships based on expert knowledge, like flow ratio); (2) An exhaustive search to evaluate all CV candidates, which is computationally expensive.

The first disadvantage restricts the economic performance of the CVs. In case that the optimal single measurement CVs cannot obtain an acceptable loss *L*, an additional optimization layer is still required. A more general form is defining functions of physical relationships *c* = *Z*(*y*) as the CVs. In the past two decades, special researches have been placed on deriving linear combinations *c* = *Hy*, where H is the combination matrix to be solved [11,14,[20], [21], [22], [23], [24]]. However, most existing methods rely on the linear process model and thus their performances are confined within the neighborhood of a fixed operating point, which further restricts their practical applications. Moreover, linear models have the assumption that the operating condition can be parametrized by several disturbance variables. In many practical problems, plantwide processes, such as the off-gas system, often define various specific operating modes/conditions, other than in terms of parametrized disturbance variables. These operating conditions are discrete and difficult to be characterized by linear models.

The second disadvantage demonstrates that the computation load should be taken into considerations. The CV selection is a combinational optimization problem, leading to an explosive increase for the computation demand when the problem scale is large. There may be numerous variables in plantwide processes, hence it is difficult to complete an exhaustive search within a limited time. To resolve this issue, the fast selecting algorithms like the branch-and-bound method were developed [42–44], making it possible to deal with large-scale processes rapidly. These algorithms can be embedded into the Skogestad's procedure to aid the search of self-optimizing CVs. For example [44], is capable of identifying the optimal single-measurement CVs based on the maximal gain rule, and [42,43] were proposed based on the local worst-case and average losses. Nonetheless, these mentioned algorithms are developed for local SOC approaches.

## 3 Enhanced CSD for chemical processes

The enhanced CSD tool presented in this paper is mainly concerned with the CV selection step embedded the full design procedure, in order to address the limitations mentioned above. Among various improved solutions concerning the mentioned limitations in Ref. [13], we emphasize and advocate the use of gSOC-plus-BAB, namely, the global SOC method [16] and a tailored BAB algorithm [42,45] for fast screening of measurements, which, in our view, are especially efficient for plantwide problems.

### 3.1 The global SOC method for CV selection

Compared with other local SOC methods, the gSOC has advantages as follows: (1) The gSOC is not restricted to linear models and its self-optimizing performance is global; (2) For the calculation of the overall system loss, discrete operating points are handled independently, while for continuous operating space, they are sampled by the Monte Carlo method. Basically, it contributes a flexible solution to the general multimode systems. A brief introduction of the gSOC is presented as follows:

The second-order Taylor series for *J* at the optimal point under a specific operating condition is expanded as (Eq. (6))

(6) $$J=J^{opt}+J_{c}^{T}e_{c}+0.5e_{c}^{T}J_{cc}e_{c}$$

where *J*_c_ and *J*_cc_ are the first-order partial derivative and the second-order Hessian matrix, respectively. *e*_c_ = *c*−*c*^opt^ describes the deviation between *c* and their optimal values. According to the optimality condition, *J*_c_ = 0 and *e*_c_ = −*Hy*_*m*_^opt^ can be obtained, *y*_m_^opt^ = *y*^opt^ + *n* is the optimal measurements with the influence of system noise. Therefore, the loss *L* can be described as the following quadratic function (Eq. (7)):

(7) $$L=J-J^{opt}=\frac{1}{2}{\left(Hy_{m}^{opt}\right)}^{T}J_{cc}\left(Hy_{m}^{opt}\right)$$

The average loss of *L*, *L*_av_ = *E*[*L*], can be derived and approximated as (Eq. (8)):

(8) $$L_{av}\approx \frac{1}{2N}\sum_{i=1}^{N}{\left(Hy_{\left(i\right)}^{opt}\right)}^{T}J_{cc}Hy_{\left(i\right)}^{opt}+\frac{1}{2}tr\left(W^{2}H^{T}J_{cc}H\right)$$

where *tr*() is the trace of a matrix, the diagonal elements of the diagonal matrix *W* are corresponding to the amplitudes of measurement noise. The right-hand side term in the above equation represents the average loss of all operating conditions and *N* is the number of total operating condition.

It is common to introduce a constraint of *HG*_y_ = *J*_*uu*_^1/2^ [16], where *G*_y_ is the input-output gain matrix at a reference point, *J*_uu_ is the Hessian matrix of *J* respect to *u*. In this case, the loss function can be simplified as (Eq. (9) and (10)):

(9) $$L_{av}=\frac{1}{2N}{\left\|YH^{T}\right\|}_{F}^{2}+\frac{1}{2}{\left\|WH^{T}\right\|}_{F}^{2}=\frac{1}{2}{\left\|\tilde{Y}H^{T}\right\|}_{F}^{2}$$

where

(10) $$Y=\left[\begin{array}{l} {\left(y_{\left(1\right)}^{opt}\right)}^{T}\\ \vdots \\ {\left(y_{\left(N\right)}^{opt}\right)}^{T} \end{array}\right],\tilde{Y}=\left[\begin{array}{l} \frac{1}{\sqrt{N}}Y\\ W \end{array}\right]$$

The final optimization problem is obtained as (Eq. (11)):

(11) $$\min_{H}L_{av}=\min_{H}\frac{1}{2}{\left\|\tilde{Y}H^{T}\right\|}_{F}^{2}s.t.\;HG_{y}=J_{uu}^{1/2}$$

which has a closed-form solution as (Eq. (12))

(12) $$H^{T}={\left({\tilde{Y}}^{T}\tilde{Y}\right)}^{-1}G_{y}{\left(G_{y}{\left({\tilde{Y}}^{T}\tilde{Y}\right)}^{-1}G_{y}\right)}^{-1}J_{uu}^{1/2}$$

In brief, the following steps are implemented to obtain the optimal combination matrix *H*:

1.3.1 Define all possible disturbance scenarios or operating conditions;
1.3.2 Solve the original optimization problem (minimize *J* in (3)), the optimal output variable *y*_(i)_^opt^ associated with all operating scenarios are obtained;
1.3.3 Construct intermediate matrices *Y* and *Ỹ* as in (10);
1.3.4 Select an operating point as the reference point (usually, the nominal point), evaluate the corresponding Hessian matrix *J*_uu_ and gain matrix *G*_y_;
1.3.5 Calculate the optimal combination matrix *H* per (12).

In the context of plantwide CSD, the above steps are suggested to replace step 1.3 of the systematic approach described in section 2, which can improve the SOC performance.

### 3.2 The BAB algorithm

The above gSOC method solves the optimal combination matrix H for given measurement subsets. However, how to select an optimal subset out of substantial measurements is also important for practical implementations. In literature, a series of BAB algorithms have been proposed as efficient tools for fast screening based on different SOC criterions. For example, the bidirectional BAB algorithm was based on the maximal minimum singular value rule [44], and the partial bidirectional BAB algorithm (PB^3^) [42] was developed for the local average loss criterion.

In the BAB algorithm, the combinational problem for selecting measurement subsets out of many is formulated as the expansion or shrinkage of branches which represent measurements being selected or discarded. The nodes of branches are evaluated against upwards and downwards pruning criteria. Branches, which satisfy either upwards or downwards pruning criteria will be fixed or removed from the candidate lists, respectively. Based on the established monotonic rule for the cost function, most non-optimal candidate subsets will be eliminated without further evaluations, so that the optimal subset can be efficiently identified.

Among various BAB variants, the PB^3^ algorithm [42], which is based on the local average loss criterion, has been proven to be successful and widely applied. The PB^3^ algorithm was further extended to the global average loss criterion (which is employed in this paper), thus it can be efficiently combined with the gSOC method to deal with large-scale problems [45]. Compared with the local average loss criterion, the matrices required by (12) are updated within the framework of the PB^3^ algorithm. The readers are referred to Ref. [42] for full descriptions of this algorithm and the code package is available via [46].

The application of the gSOC-plus-BAB strategy typically produces a Pareto front in terms of the economic indicator, *L*_av_, and the size of measurement subset, *n*_y_. Therefore, it is always required to make a trade-off between the two factors, as the increase of *n*_y_ may result in reduced level of economic benefits. For a given *n*_y_, the algorithm is also able to generate the first several best subsets by ranking the economic losses. These candidate solutions will be retained for further nonlinear model evaluations as well as dynamic validations. This consideration is the same as [13] for the sake of practical performance assurance.

## 4 The industrial off-gas system

### 4.1 Process descriptions

**Fig. 2:**
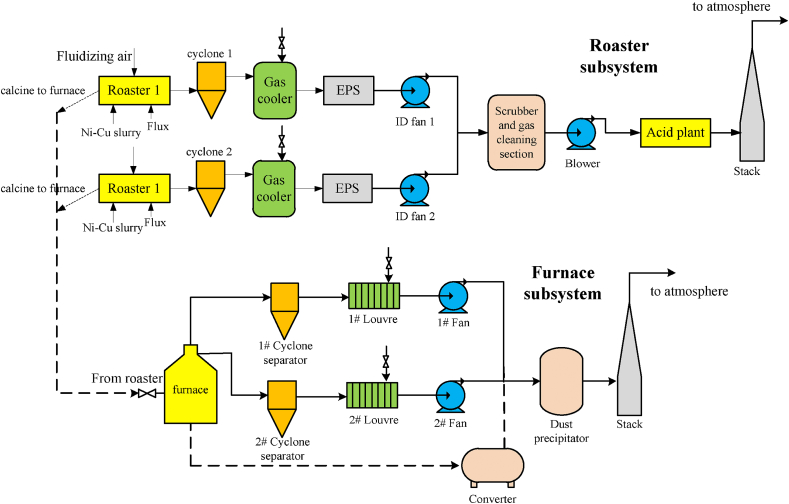
The industrial off-gas system.

The industrial off-gas plant, the Xstrata Nickels Sudbury Smelter cleaning system [26,27] is investigated in this paper. The off-gas plant is composed of a roaster subsystem and a furnace subsystem, each of which contains two parallel operating lines, as illustrated in Fig. 2.

In the fluidized bed roaster, the copper-nickel slurry from mineral processing plants reacts with the fed fluidizing air, which generates a lot of SO_2_. The off-gas from the roaster firstly enters a cyclone and then is cooled from 650 to 680 to 300–350 °C by the water spray. The electro-static precipitator (ESP) processes the gases, followed by an induced draft (ID) fan draws the gases to combine with the other parallel roaster line. The combined gases go through the cleaning train to get rid of the dust. Finally, the acid plant blower transports the dried dust free gases to the wet acid plant, where sulfur dioxide is converted to sulfuric acid.

The hot products from the roaster are fed into the furnace, where reactions occur with carbon electrodes and a large amount of CO 2 is produced. The high-temperature off-gas generated from the furnace, enters the cyclone separator to separate solid particle via two pipelines from the top of the calcinator. Then the gases flow into the louver fin heat exchanger to be cooled down and converge after the fan from each pipeline. Besides, the product of the calcinatory enters the converter and reacts with oxygen to generate iron slag as well as other components. The three gases converge into the Cottrell electric dust precipitator and emitted through the chimney after purification.

### 4.2 Mathematical models

Benchmark models for the off-gas system, both steady-state and dynamic versions, have been developed in Ref. [34]. Basically, the models are built based on mass, momentum and energy conservation laws. A series of reasonable assumptions are also introduced for model simplifications. For better understanding the new control configuration, a sketch of the model equations is listed in the following, more details are available in Ref. [34].

#### 4.2.1 Roaster subsystem model

Around the roaster unit, the model equations for the pressure, temperature, and mass flowrate, are (Eqs. 13–15)

(13) $$\frac{dP_{R}}{dt}=\frac{c_{p}R}{V_{R}\left(c_{p}-R\right)}\left[\left(T_{0}+\frac{h_{R}}{c_{p}}\right)W_{\text{in}}-T_{R}W_{R}\right]\text{,}$$

(14) $$\frac{dT_{R}}{dt}=\frac{RT_{R}}{P_{R}V_{R}\left(c_{p}-R\right)}\left[\left(c_{p}T_{0}+h_{R}\right)W_{\text{in}}-\left(c_{p}-R\right)T_{R}W_{\text{in}}-RW_{R}T_{R}\right]\text{,}$$

(15) $$\frac{dW_{R}}{dt}=\frac{1}{L_{R-C}}\left[\left(P_{R}-P_{C}\right)a-\frac{k_{C}}{\rho }W_{R}^{2}\right]$$

where all notations are explained in Table 1. The equations do not indicate the index of the two roaster lines because their models are the same. The symbols will, however, be labelled with indices as subscripts in later analysis when necessary.

**Table 1:** Notations for the off-gas system.

*a*	cross-sectional area of the ducts between the units	m^2^
*A*_leak_	in-leakage area	m^2^
*C*_d_	in-leakage coefficient	kg/(s m^2^ Pa^0.5^)
*c*_p_	specific heat	J/(kg °C)
*c*_p0_	specific heat of air	J/(kg °C)
*c*_pwl_	average specific heat of water before boiling	J/(kg °C)
*c*_pwg_	average specific heat of water vapor	J/(kg °C)
*h*_R_	enthalpy of the roasting reaction per unit mass of off-gas production	J/kg
*h*_vap_	enthalpy of water vaporization	J/kg
*h*_SC_	enthalpy loss in scrubber cooling per unit mass of off-gas entering the scrubber	J/kg
*k*	coefficient of momentum loss due to friction	N/(kg/s)^2^ or N/(kg/s)
*K*	coefficient that accounts for compressibility of gas	
*L*	length of gas flow path	m
*N*	fan speed	rpm
*O*	louver opening	
*p*	price of electricity	$/J
*p*_f_	price of feed	$/J
*P*	Pressure	Pa
*P*_atm_	atmospheric pressure	Pa
*R*	gas constant	J/(kg °C)
*t*	Time	s
*T*	Temperature	°C
*T*_0_	room temperature	°C
*T*_bo_	water boiling temperature	°C
*V*	Volume	m^3^
*W*	mass flow rate	kg/s
*W*_in_	off-gas mass flow rate coming into the roaster freeboard	kg/s
*W*_rf_	mass flow rate of roaster feeds that become off-gas	kg/s
*x*_H2O_	mass fraction of H_2_O in off-gas	
*Z*	fan or blower vane position	%
*α*	coefficient of pressure change due to vane position change	Pa/%
*β*	coefficient relating pressure in fan or blower to flow rate	Pa/(kg/s)^2^
*γ*	power index relating momentum loss to mass flow rate due to friction	
*ε*	fan efficiency	
*ρ*	Density	kg/m^3^
Subscripts		
AC	wet acid plant	
B	blower or blower outlet	
R	roaster or roaster outlet	
C	cyclone or cyclone outlet	
Conv	converter off-gas	
G	gas cooler or gas cooler outlet	
E	electro-static precipitator or electro-static precipitator outlet	
IDfan	ID fan or outlet (roaster)	
Fan	fan or fan outlet (furnace)	
F	furnace or furnace outlet	
fan-Si	from fan to scrubber inlet	
Lv	louver or louver outlet	
Si	scrubber inlet	
S	scrubber and gas cleaning section or outlet of this section	
W	water added in the gas coolers	

The cyclone takes the following similar form (Eqs. (16)-(18))

(16) $$\frac{dP_{C}}{dt}=\frac{c_{p}R}{V_{C}\left(c_{p}-R\right)}\left[T_{R}W_{R}-T_{C}W_{C}\right]$$

(17) $$\frac{dT_{C}}{dt}=\frac{RT_{C}}{P_{C}V_{C}\left(c_{p}-R\right)}c_{p}\left(T_{R}-T_{C}\right)W_{R}-RT_{C}\left(W_{C}-W_{R}\right)$$

(18) $$\frac{dW_{C}}{dt}=\frac{1}{L_{C-G}}\left[\left(P_{C}-P_{G}\right)a-k_{G}W_{C}^{2}\right]$$

For the gas cooler, the equations are (Eqs. (19)-(21))

(19) $$\frac{dP_{G}}{dt}=\frac{c_{p}R}{V_{G}\left(c_{p}-R\right)}\times \{T_{C}W_{C}-T_{G}\left(W_{G}-W_{w}\right)\frac{W_{w}}{c_{p}}\left[c_{\text{pwl}}\left(T_{0}-T_{\text{bo}}\right)-h_{\text{vap}}+c_{\text{pwg}}\left(T_{\text{bo}}-T_{G}\right)\right]\}$$

(20) $$\begin{array}{l} \frac{dT_{G}}{dt}=\frac{RT_{G}}{P_{G}V_{G}\left(c_{p}-R\right)}\{c_{p}\left(T_{C}-T_{G}\right)W_{C}+W_{w}[c_{\text{pwl}}\left(T_{0}-T_{\text{bo}}\right)-h_{\text{vap}}\\ +c_{\text{pwg}}\left(T_{\text{bo}}-T_{G}\right)]+RT_{G}\left(W_{C}+W_{w}-W_{G}\right)\} \end{array}$$

(21) $$\frac{dW_{G}}{dt}=\frac{1}{L_{G-E}}\left[\left(P_{G}-P_{E}\right)a-k_{E}W_{G}^{2}\right]$$

The ESP is described by (Eqs. (22)-(23)

(22) $$\frac{dP_{E}}{dt}=\frac{c_{p}R}{V_{E}\left(c_{p}-R\right)}\left(T_{G}W_{G}-T_{E}W_{E}\right)$$

(23) $$\frac{dT_{E}}{dt}=\frac{RT_{E}}{P_{E}V_{E}\left(c_{p}-R\right)}\left[c_{\text{pE}}\left(T_{G}-T_{E}\right)W_{G}-RT_{E}\left(W_{E}-W_{G}\right)\right]$$

The pressure drop through the ID fan is related to the vane position as (Eq. (24))

(24) $$P_{E}-P_{\text{IDfan}}=\alpha_{\text{fan}}Z_{\text{fan}}+\beta_{\text{fan}}W_{E}^{2}$$

For the scrubber, the equations are (Eqs. (25)-(27)

(25) $$\frac{dW_{\text{fan}}}{dt}=\frac{1}{L_{\text{fan}-\text{Si}}}\left[\left(P_{\text{fan}}-P_{\text{Si}}\right)a-k_{\text{Si}}W_{\text{fan}}^{2}\right]\text{,}$$

(26) $$\frac{dP_{\text{Si}}}{dt}=\frac{c_{p}R}{V_{\text{fan}-\text{Si}}\left(c_{p}-R\right)}\left(T_{{\text{fan}}_{1}}W_{{\text{fan}}_{1}}+T_{{\text{fan}}_{2}}W_{{\text{fan}}_{2}}-T_{\text{Si}}W_{\text{Si}}\right)\text{,}$$

(27) $$\frac{dT_{\text{Si}}}{dt}=\frac{RT_{\text{Si}}}{P_{\text{Si}}V_{\text{fan}-\text{Si}}\left(c_{p}-R\right)}\left[c_{p}\left(T_{{\text{fan}}_{1}}-T_{\text{Si}}\right)W_{{\text{fan}}_{1}}+c_{p}\left(T_{{\text{fan}}_{2}}-T_{\text{Si}}\right)W_{{\text{fan}}_{2}}-RT_{\text{Si}}\left(W_{\text{Si}}-W_{{\text{fan}}_{1}}-W_{{\text{fan}}_{2}}\right)\right]$$

The model equations for the scrubber and the gas cleaning section are (Eqs. (28)-(30)

(28) $$\frac{dW_{\text{Si}}}{dt}=\frac{1}{L_{S}}\left(P_{\text{Si}}-P_{S}\right)a-k_{S}W_{\text{Si}}^{\gamma }$$

(29) $$\frac{dP_{S}}{dt}=\frac{c_{p}R}{V_{S}\left(c_{p}-R\right)}\left[\left(T_{\text{Si}}-\frac{h_{\text{SC}}}{c_{\text{ps}}}\right)W_{\text{Si}}-T_{S}W_{S}\frac{1}{\left(1-x_{H2O}\right)}\right]$$

(30) $$\frac{dT_{S}}{dt}=\frac{RT_{S}}{P_{S}V_{S}\left(c_{p}-R\right)}\left[\left(c_{p}T_{\text{Si}}-h_{\text{SC}}\right)W_{\text{Si}}-\left(c_{\text{ps}}-R\right)T_{S}W_{Si}\right]-RT_{S}\frac{W_{S}}{1-x_{H2O}}$$

The pressure drop through the blower is similar to the ID fan, as (Eq. (31))

(31) $$P_{B}-P_{S}=\alpha_{B}Z_{B}+\beta_{B}W_{S}^{\gamma }$$

Finally, the following equation describes the acid plant (Eq. (32))

(32) $$L_{\text{AC}}\frac{dW_{B}}{dt}=\left(P_{B}-P_{\text{atm}}\right)a-k_{\text{AC}}W_{B}^{\gamma }$$

#### 4.2.2 Furnace subsystem model

The modeling equations for the furnace are given by (Eqs. (33)-(35)

(33) $$\frac{dP_{F}}{dt}=\frac{c_{p}R}{V_{F}\left(c_{p}-R\right)}\left[W_{\text{CO}}\left(T_{\text{CO}}+\frac{h_{\text{CO}/\text{CO}2}}{c_{p}}\right)+W_{\text{air}}T_{0}-W_{F}T_{F}\right]$$

(34) $$\frac{dT_{F}}{dt}=\frac{RT_{F}}{P_{F}V_{F}\left(c_{p}-R\right)}[W_{\text{CO}}\left(c_{p}T_{\text{CO}}+h_{\text{CO}/\text{CO}2}\right)+W_{\text{air}\;}c_{p}T_{0}-\left(W_{\text{CO}}+W_{\text{air}\;}\right)\left(c_{p}-R\right)T_{F}-W_{F}RT_{F}]$$

(35) $$\frac{dW_{F}}{dt}=\frac{1}{L_{C}}\left[\left(P_{F}-P_{C}\right)a-\frac{k_{C}}{\rho }W_{F}^{2}\right]$$

The model equations for the scrubber and the gas cleaning section are (Eq. (36))

(36) $$W_{\text{air}\;}=C_{d}A_{\text{leak}\;}\sqrt{P_{\text{atm}}-P_{F}}$$

For the louver, the equations are (Eqs. (37)-(38)

(37) $$\frac{dP_{\text{lv}}}{dt}=\frac{c_{p}R}{V_{\text{lv}}\left(c_{p}-R\right)}\left[W_{C}T_{C}+W_{\text{air}}T_{0}-W_{\text{lv}}T_{\text{lv}}\right]$$

(38) $$\frac{dT_{\text{lv}}}{dt}=\frac{RT_{\text{lv}}}{P_{\text{lv}}V_{\text{lv}}\left(c_{p}-R\right)}[W_{C}c_{p}T_{C}+W_{\text{lv}-\text{air}}c_{p}T_{0}-(W_{C}+W_{\text{lv}-\text{air}}\left(c_{p}-R\right)T_{\text{lv}}-W_{\text{lv}}RT_{\text{lv}}]$$

The fan model in the furnace is expressed as (Eq. (39))

(39) $$P_{\text{fan}-\text{out}}-P_{\text{fan}-\text{in}}=\alpha_{\text{fan}}N_{\text{fan}}^{2}$$

Various model parameters involved from (13) to ()(13) to (39)(13) to (39) are not listed here for brevity, which can be conveniently found in Ref. [34]. The systematical models developed above provide an excellent benchmark testbed for the industrial off-gas processes. Although the models are approximations to the real plants, they have been validated and behave reasonably well in most scenarios. Therefore, studies on the benchmark model would be valuable and reliable for the off-gas industry.

### 4.3 Operational objectives

In general, there are two operating modes for the off-gas system: a fixed production rate (mode I) and maximization of the production rate (mode II). In this paper, however, we will focus on the first mode to seek for economic improvements using measurement combinations. In the latter case, which is typically determined by some bottleneck variables, the optimal operation strategies have been given in Refs. [27,33], respectively. No differences will be discovered for mode II, as the bottleneck variables are all single physical variables.

The operational objectives of the two subsystems can be considered separately, which are relatively independent. In mode I, the economic objectives for the two subsystems are formulated as (Eq. (40) and (41)) [34].

(40) $$J^{R}=\left(P_{\text{IDfan}1}-P_{E1}\right)W_{F1}+\left(P_{\text{IDfan}2}-P_{E2}\right)W_{F2}+\left(P_{B}-P_{S}\right)W_{B}$$

(41) $$J^{F}=\left(P_{\text{fan}1}-P_{lv1}\right)W_{\text{fan}1}+\left(P_{\text{fan}2}-P_{lv2}\right)W_{\text{fan}2}$$

where *J*^R^ and *J*^F^ [Pa·kg/s] relate to the economic cost [$/s] by multiplying a constant factor, thus they can be equivalently minimized.

The definitions of possible disturbance scenarios are listed in Table 2 and 3 for the roaster and furnace subsystems, respectively. Besides, the process constraints that must be respected are summarized in Table 4 and 5. One notes that one critical aspect is to maintain the pressure in the system negative, to prevent gas leakage into the atmosphere [34].

**Table 2:** Disturbances for the roaster.

	Description	Nominal (disturbance)
D_R1_	inlet gas flow rate of roaster 1 and 2, *W*_in1_ and *W*_in2_ [kg/s]	18 + 18 (−10%)
D_R2_	inlet gas flow rate of roaster 1 and 2, *W*_in1_ and *W*_in2_ [kg/s]	18 + 18 (+10%)
D_R3_	inlet gas flow rate of roaster 1, *W*_in1_ [kg/s]	18 (−10%)
D_R4_	inlet gas flow rate of roaster 1, *W*_in1_ [kg/s]	18 (+10%)
D_R5_	inlet gas flow rate of roaster 2, *W*_in2_ [kg/s]	18 (−10%)
D_R6_	inlet gas flow rate of roaster 2, *W*_in2_ [kg/s]	18 (+10%)
D_R7_	effective in-leakage area in roaster 1, *A*_leak11_ [m^2^]	0.0120 (+50%)
D_R8_	effective in-leakage area in roaster 1 to ESP 1 inlet, A_leak12_ [m^2^]	0.0190 (+50%)
D_R9_	effective in-leakage area in ESP 1, A_leak13_ [m^2^]	0.0590 (+50%)
D_R10_	effective in-leakage area in roaster 2, A_leak21_ [m^2^]	0.0110 (+50%)
D_R11_	effective in-leakage area in roaster 2 to ESP 2 inlet, *A*_leak22_ [m^2^]	0.0530 (+50%)
D_R12_	effective in-leakage area in ESP 2, A_leak23_ [m^2^]	0.0350 (+50%)
D_R13_	effective in-leakage area from ID fan duct to scrubber inlet, *A*_leak31_ [m^2^]	0.0820 (+50%)
D_R14_	effective in-leakage area in gas cleaning section, *A*_leak32_ [m^2^]	0.1300 (+50%)
D_R15_	effective in-leakage area in blower inlet, *A*_leak33_ [m^2^]	0.0220 (+50%)
D_R16_	room temperature, T_0_ [K]	273 (−30)
D_R17_	room temperature, T_0_ [K]	273 (+30)

**Table 3:** Disturbances for the furnace.

	Description	Nominal (disturbance)
D_F1_	Coke added to the furnace, *W*_coke_ [kg/s]	2 (+20%)
D_F2_	Coke added to the furnace, *W*_coke_ [kg/s]	2 (−10%)
D_F3_	Equivalent CO temperature in the furnace, *T*_co_ [K]	1573.15 (+200)
D_F4_	Equivalent CO temperature in the furnace, *T*_co_ [K]	1573.15 (−200)
D_F5_	Flow rate from converters, *W*_conv_ [kg/s]	300 (+20%)
D_F6_	Flow rate from converters, *W*_conv_ [kg/s]	300 (−20%)
D_F7_	Converter outlet temperature, *T*_conv_ [K]	473.15 (+100)
D_F8_	Converter outlet temperature, *T*_conv_ [K]	473.15 (−100)
D_F9_	Effective in-leakage area in the furnace, *A*_fleak_ [m^2^]	1.50 (+50%)
D_F10_	Room temperature, *T*_0_ [K]	273 (+30)
D_F11_	Room temperature, *T*_0_ [K]	273 (−30)

**Table 4:** Process constraints for the roaster.

Description	bounds
roaster 1 freeboard pressure *P*_R1_ [Pa]	<-1000
roaster 2 freeboard pressure *P*_R2_ [Pa]	<-1000
cyclone 1 pressure *P*_C1_ [Pa]	<0
cyclone 2 pressure *P*_C2_ [Pa]	<0
gas cooler 1 pressure *P*_G1_ [Pa]	<0
gas cooler 2 pressure *P*_G2_ [Pa]	<0
gas cooler 1 temperature *T*_G1_ [K]	<643.15
gas cooler 2 temperature *T*_G2_ [K]	<643.15
water to gas cooler 1 *W*_w1_ [kg/s]	>0
water to gas cooler 2 *W*_w2_ [kg/s]	>0
electro-static precipitator (ESP) 1 pressure *P*_E1_ [Pa]	<0
electro-static precipitator (ESP) 2 pressure *P*_E2_ [Pa]	<0
ID fan 1 outlet pressure *P*_IDfan1_ [Pa]	<-200
ID fan 2 outlet pressure *P*_IDfan2_ [Pa]	<-200
ID fan 1 vane position *Z*_F1_ [%]	[0 100]
ID fan 2 vane position *Z*_F2_ [%]	[0 100]
Scrubber inlet pressure *P*_Si_ [Pa]	<0
gas cleaning section pressure *P*_S_ [Pa]	<0
blower vane position *Z*_B_ [%]]	[0 100]

**Table 5:** Process constraints for the furnace.

Description	bounds
Calcinator pressure *P*_f_ [Pa]	< −25
Cyclone separator pressure *P*_c_ [Pa]	< −25
Cooling unit 1 pressure *P*_lv1_ [Pa]	<0
Cooling unit 2 pressure *P*_lv2_ [Pa]	<0
Fan 1 outlet pressure *P*_fan1_ [Pa]	<0
Fan 2 outlet pressure *P*_fan2_ [Pa]	<0
Dust precipitator pressure *P*_iCt_ [Pa]	<0
Calcinator temperature *T*_f_ [K]	<923.15
Cooling unit 1 temperature *T*_lv1_ [K]	<643.15
Cooling unit 2 temperature *T*_lv2_ [K]	<643.15
Dust precipitator temperature *T*_iCt_ [K]	<643.15
Cooling unit 1 opening *O*_1_ [%]	[0 100]
Cooling unit 2 opening *O*_2_ [%]	[0 100]
Fan 1 speed *N*_fan1_ [rpm]	<1500
Fan 2 speed *N*_fan2_ [rpm]	<1500

## 5 Enhanced CSD results

Previously, the Skogestad's systematical CSD procedure [13] has been followed tightly to configure the control systems for this benchmarked off-gas process [27,33,34]. Therefore, in the following we will not repeatedly present the whole systematical procedure. In contrast, the obtained CSD results, particularly for the selected self-optimizing CVs based on the SOC principle will be briefly reviewed, and our main improvements (measurement combinations as CVs) will be highlighted to show the new contributions.

### 5.1 CSD for the roaster subsystem

#### 5.1.1 Available results

There are five degrees of freedom for the roaster lines: water addition to the gas coolers, *W*_w1_ and *W*_w2_, the ID fan vane positions, *Z*_fan1_ and *Z*_fan2_ and the blower vane position *Z*_B_. To select the primary CVs, rigorous analysis indicated that four variables are the active constraints:

Roaster 1 freeboard pressure (upper bound) (Eq. (42)):

(42) $$P_{R1}=-1000\;\left[Pa\right]$$

Roaster 2 freeboard pressure (upper bound) (Eq. (43)):

(43) $$P_{R2}=-1000\;\left[Pa\right]$$

Gas cooler 1 temperature (upper bound) (Eq. (44)):

(44) $$T_{G1}=643.15\;\left[K\right]$$

Gas cooler 2 temperature (upper bound) (Eq. (45)):

(45) $$T_{G2}=643.15\;\left[K\right]$$

Control of the above four active constraints consumes four degrees of freedom. Then, only one free variable is left to select the additional self-optimizing CV. The identified promising CVs using single measurements (*n*_y_ = 1) [33] are given in Table 6. It is worth mentioning that their setpoints were re-optimized such that feasibility is ensured, following the robust setpoint method [47]. In this problem, the feasibility is most likely to be violated for constraints where *P*_IDfan1_ and *P*_IDfan2_ should be less than −200 [Pa] for all disturbances. Based on the steady state evaluations via nonlinear process models, the final chosen self-optimizing CV by Ref. [33] is the scrubber inlet pressure, *P*_si_ (setpoint: −866.98 [Pa]), whose worst and average economic losses are evaluated to be 0.894 and 0.407, respectively.

**Table 6:** Candidate CVs for the roaster and their economic losses.

*n*_y_	CV	robust setpoint	maximal loss	average loss
1	*P*_si_	−866.98	0.894	0.407
	*P*_fan2_	−308.78	0.685	0.428
	*P*_fan1_	−314.22	0.704	0.441
2	*c*_21_: 0.494*P*_IDfan1_+0.506*P*_IDfan2_	−257.04	0.356	0.190
	*c*_22_: 0.0413*P*_si_ +0.959*T*_si_	12.894	0.474	0.199
	*c*_23_: 0.906*P*_si_−0.094*P*_s_	253.25	0.474	0.200
3	*c*_31_: 0.013*P*_si_ −0.002*P*_b_+0.9952*Z*_b_	3.56	0.474	0.184
	*c*_32_: 0.009*P*_s_−0.415*W*_s_ + 0.576*Z*_b_	2.69	0.474	0.184
4	*c*_41_: 0.0024*P*_si_−0.264*W*_si_−0.0011*P*_b_+0.7325*Z*_b_	0.361	0.737	0.209
	*c*_42_: 0.0015*P*_si_+0.0018*P*_s_−0.0015*P*_b_+0.9952*Z*_b_	0.50	0.724	0.207
…	*…*			
12	*c*_full_: *Hy*^R^_full_	0.3634	0.377	0.183

**Fig. 3:**
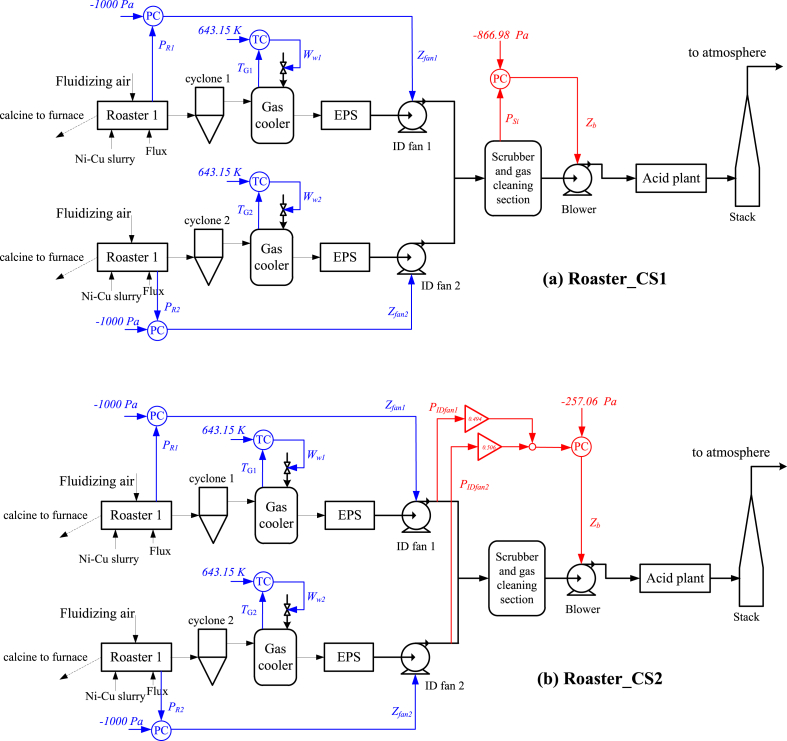
Control structures for the roaster subsystem: (a) results in Ref. [33]; (b) reconfiguration using the enhanced design.

With the four active constraints and one unconstrained self-optimizing CV, the control configurations for the control layers/loops following the bottom-up design are illustrated in Fig. 3 (a), where the pairings between the CVs and MVs are determined using the RGA analysis. For all the five single-input-single-output control loops, the PID controllers are adopted and tuned using the SIMC tuning rule [48]. Dynamic simulations have been carried out to validate the control structure, more details can be found in Ref. [33].

#### 5.1.2 Enhanced reconfiguration

In the following, we reconsider the step for identification of the self-optimizing CV, using measurement combinations. Totally, there are 58 measurements in the roaster subsystem. However, some of them can be preliminarily removed via steady-state controllability analysis to shrink the search of candidate CVs. Actually, the poor controllability (small gains from *Z*_b_ to the outputs, *G*_y_) will indeed lead to large economic loss, because the economic loss *L* is proportional to the inverse of *HG*_y_ as conveyed by their quantitative relationship. Furthermore, the search process will become ill-defined when the associated *G*_y_ is singular. Finite difference is considered to evaluate the gains at the nominal point, assuming that the active constraints are already perfectly controlled using the four MVs. By eliminating those relative gains less than 1E-5, we are left with the following 13 measurements for further analysis (Eq. (46)):

(46) $$y_{full}^{R}={\left[P_{si}\;T_{si}\;W_{si}\;P_{s}\;T_{s}\;W_{s}\;Z_{f1}\;Z_{f2}\;P_{f1}\;P_{f2}\;P_{b}\;T_{b}\;Z_{b}\right]}^{T}$$

For all the 18 operating conditions (nominal+17 disturbances), optimizations are performed to obtain the optimal values of *y*^R^_full_ (see Ref. [33] for numerical results) to formulate the matrix *Y* in the gSOC method. Other sensitivities required by the approach are all evaluated at the nominal condition. Even with the 13 measurements, it is not a trial task to find the optimal measurement subset with different sizes. Therefore, the gSOC based BAB algorithm is applied to search promising subsets when *n*_y_ varies from 2 to 13. This selection process is almost instantaneously accomplished by the BAB algorithm implemented on a laptop with Intel-i7 CPU, 16 GB RAM. The results are summarized in Table 6. Similarly, we also adjust the setpoint of all CVs using the robust setpoint method in Ref. [47], to fulfill the feasibility regarding *P*_IDfan1_ and *P*_IDfan2_.

The best CV for *n*_y_ = 2 is identified as *c*_21_ = 0.494*P*_IDfan1_+0.506*P*_IDfan2_ (setpoint: −257.04 [Pa]), whose worst and average losses are evaluated to be 0.356 and 0.190, respectively. The losses correspond to reductions by 60.2% and 53.3%, compared with the previous result (control *P*_si_ = −866.98 [Pa] [33]). We note that *c*_21_ is composed by *P*_IDfan1_ and *P*_IDfan2_, which are exactly the constrained variables that are likely to violate their upper bounds. In some sense, controlling their (approximately average) combination with a setpoint of −257.04 can be regarded as a back-off strategy, which is advantageous to increase the operational confidence. The second and third best CVs, *c*_22_ = 0.0413 *P*_si_+0.959 *T*_si_ and *c*_23_ = 0.906 *P*_si_−0.094 P, use different measurements and lead to larger losses.

It turns out that the gained economic benefit by further increasing *n*_y_ is limited. The best CVs for *n*_y_ = 3 reduce very little economic loss compared with *n*_y_ = 2, as shown in Table 6. Surprisingly, the loss even increases in the case of *n*_y_ = 4, which is found to be caused by the discrepancy between the gSOC criterion and the nonlinear loss evaluations, and additionally the robust setpoint adjustment. Furthermore, we have tested that by using all measurements, *c*^R^_full_ = *Hy*^R^_full_, the average loss is 0.183, which is only slightly smaller than the loss of *c*_21_, but the form of *c*^R^_full_ is clearly much more complicated. Through the above extensive analysis, we would like to finally recommend *c*_21_ = 0.494*P*_IDfan1_+0.506*P*_IDfan2_ (setpoint: −257.04 [Pa]) as the new self-optimizing CV for the roaster, owing to the following observations:

1. Among all identified candidates, *c*_21_ makes the best trade-off between the CV complexity and the economic benefit. Compared with the original CV in Ref. [33], *P*_si_, the mathematical form of *c*_21_ is only slightly complicated, but the economy is improved a lot (Fig. 4);

**Fig. 4:**
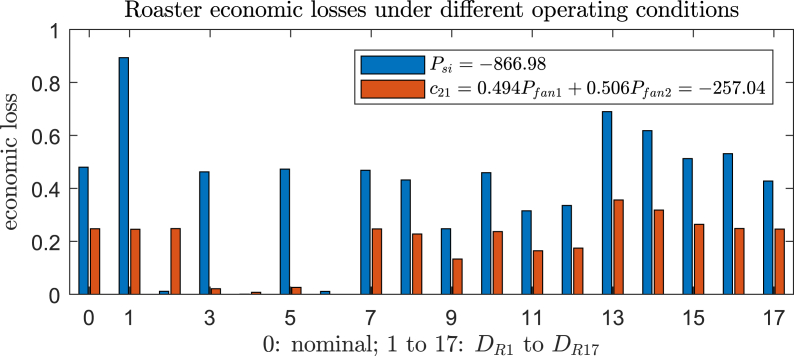
Economic losses by controlling *P*_si_ and *c*_21_ under different operating conditions.
2. The two constrained variables, *P*_IDfan1_ and *P*_IDfan2_, are directly incorporated in *c*_21_, thus the scheme could be trustworthy by field operators (in the sense of practical operation);
3. The dynamic behavior between the input (*Z*_b_) and *c*_21_ is satisfactory, as will be discovered in the subsequent dynamic simulations.

**Dynamic validations.** The proposed control reconfiguration is thus to replace the previous control loop *Z*_b_↔*P*_si_ with the new one, *Z*_b_↔*c*_21_, while the four other active constraint control loops are preserved, as indicated by Fig. 3 (b). The PID parameters for the new control loop are easily tuned using the SIMC rule. For comparison purpose, the original control structure in Ref. [33] is referred as Roaster_CS1 and ours is referred as Roaster_CS2.

**Fig. 5:**
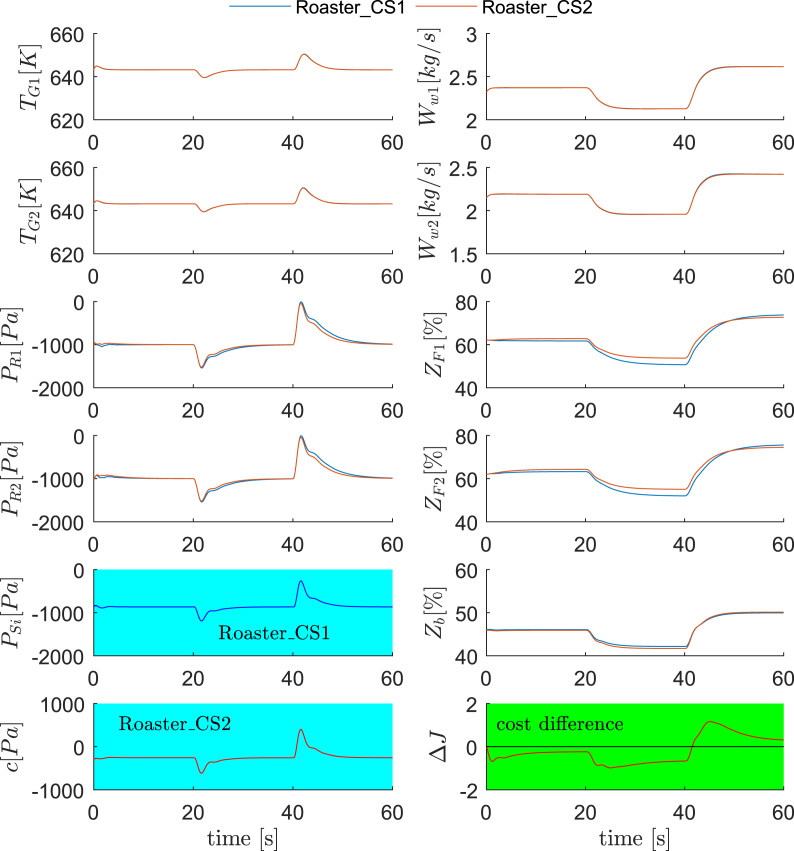
Dynamic comparisons between Roaster_CS1 and Roaster_CS2 (Scenario 1). 0–20 s: nominal condition; 20–40 s: D_R1_; 40–60 s: D_R2_.

**Fig. 6:**
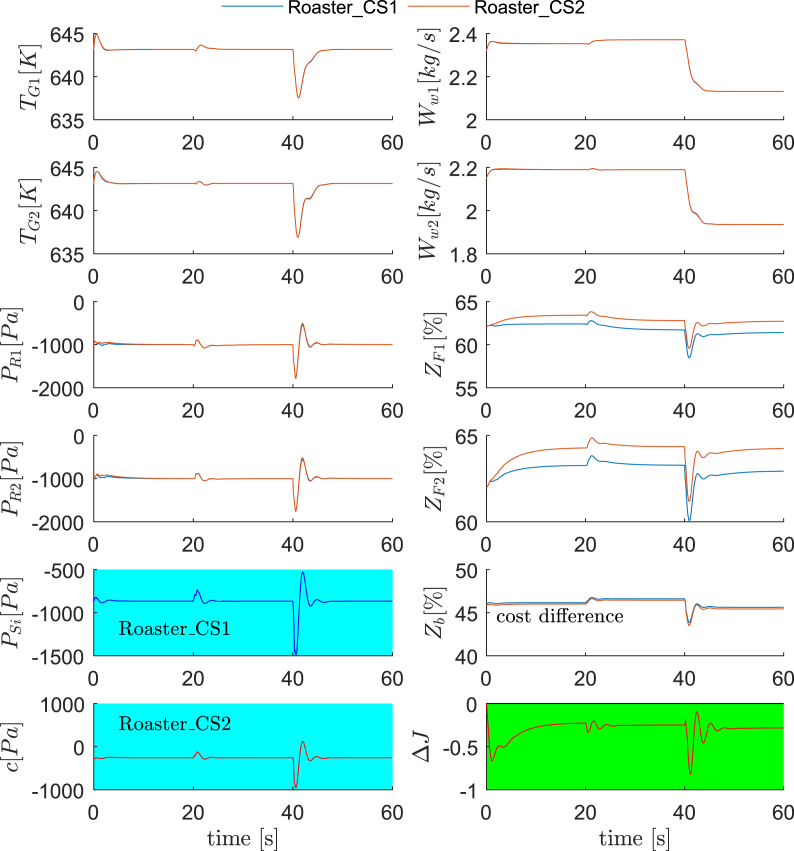
Dynamic comparisons between Roaster_CS1 and Roaster_CS2 (Scenario 2). 0–20 s: D_R7_; 20–40 s: D_R15_; 40–60 s: D_R16_.

The dynamic simulations for Roaster_CS1 and Roaster_CS2 are compared under Scenario 1 and Scenario 2, each of which contains three changing disturbance conditions for every 20 s. In Scenario 1, the operating conditions are the nominal, D_R1_ and D_R2_, respectively. In Scenario 2, the operating conditions are D_R7_, D_R15_ and D_R16_, respectively. The dynamic results are summarized in Fig. 5 and 6, plotting all controlled variables and manipulated variables, as well as the cost differences between Roaster_CS1 and Roaster_CS2. As clearly shown, the dynamic response of Roaster_CS2, in terms of the regulatory control quality, is comparable to Roaster_CS1, if not better than. However, the economic performance of Roaster_CS2 is evidently improved, where a negative Δ*J* stands for improvements (for 40–60 s in Fig. 5, Roaster_CS2 is worse than Roaster_CS1). Furthermore, one can confirm that the converged Δ*J* in each disturbance condition in Fig. 5 and 6 agree with the steady state results in Fig. 4.

The above simulation studies verify the fact that our new solution does not upset the closed-loop response compared with the one in Ref. [33]. However, the overall economic performance is effectively improved using very simple reconfigurations.

### 5.2 CSD for the furnace subsystem

#### 5.2.1 Available results

Now consider the furnace subsystem, which has four degrees of freedom: Louver 1 vane opening *O*_1_, Louver 2 vane opening *O*_2_, Fan 1 rotation speed, *N*_fan1_, and Fan 2 rotation speed, *N*_fan2_. Among the process constraints of furnace in Table 5, the following three active constraints are identified:

Calcinator temperature (upper bound) (Eq. (47)):

(47) $$T_{f}=923.15\;\left[K\right]$$

Cooling unit 1 temperature (upper bound) (Eq. (48)):

(48) $$T_{lv1}=643.15\;\left[K\right]$$

Cooling unit 2 temperature (upper bound) (Eq. (49)):

(49) $$T_{lv2}=643.15\;\left[K\right]$$

Again, it leaves one degree of freedom for the selection of self-optimizing CV. Similarly, the Skogestad's systematical procedure was followed in Ref. [27], and the authors finally suggested to select, among 43 measurements in the furnace, one of the two fan speed, *N*_fan1_ or *N*_fan2_ (setpoint: 522.08 [rpm]) as the self-optimizing CV, which was said to give the minimal economic loss for all possible configurations via controlling single measurements [27].

**Fig. 7:**
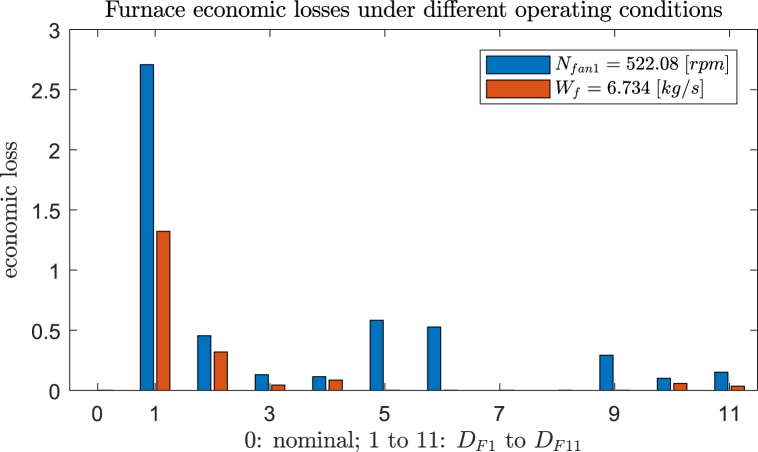
Furnace economic losses by controlling *N*_fan1_ under different operating conditions.

**Fig. 8:**
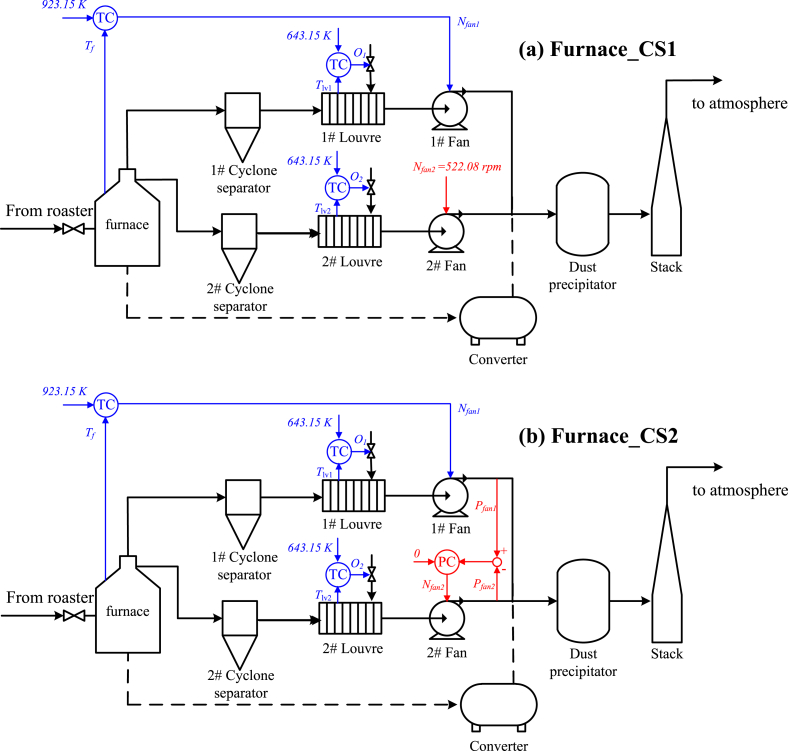
Control structures for the furnace subsystem: (a) results in Ref. [27]; (b) reconfiguration using the enhanced design.

In the following, without loss of generality, we consider the selection of *N*_fan2_ as an illustration. Actually, this is an open loop policy, as *N*_fan2_ is the manipulated variable. We calculated that the maximal and average economic losses for this scheme are 2.708 and 0.422, respectively, corresponding to 6.6% and 42.7% of the nominal cost, which are unfortunately quite large. The detailed steady state closed-loop losses by fixing *N*_fan2_ = 522.08 [rpm] for all disturbance conditions are shown in Fig. 7. Based on this selection, the overall control structure for the furnace designed by Ref. [27] is illustrated in Fig. 8 (a), where the three feedback control loops for active constraints are paired as *N*_fan1_↔*T*_f_, *O*_1_↔T_lv1_ and *O*_2_↔*T*_lv2_.

#### 5.2.2 Enhanced reconfiguration

The same steps as in the roaster case implementing the gSOC-plus-BAB method are applied to the furnace. Therefore, for the sake of brevity, only the most relevant results are presented and discussed in the following.

In the case of single measurements (*n*_y_ = 1), we identified better options recommended by the BAB algorithm. Our best CV is the total flow of gas leaving the furnace, *W*_f_, with the setpoint of 6.734 kg/s. Using nonlinear model evaluations, this scheme results in maximal and average losses of 1.322 and 0.157, respectively, which results in substantial improvements compared to fixing *N*_fan2_ as shown in Fig. 7. The suboptimality by Ref. [27] again demonstrates the advantage of the gSOC-plus-BAB design method, namely, an automated flow to identify performance-guaranteed self-optimizing CVs, which could be easily missed by a heuristic search.

By increasing *n*_y_, we obtain even more interesting outcomes. As long as *n*_y_ = 2, zero losses are achieved for all disturbance conditions, which attain perfect self-optimizing control. The derived CV is either *c*_1_ = *P*_fan1_−*P*_fan2_ or *c*_2_ = *P*_lv1_−*P*_lv2_, both of whose setpoints are 0 as computed by the gSOC method. The physical implication of the obtained CVs is to maintain the pressures of the two parallel lines in the furnace subsystem equal, which seems an intuitive rule that can be understood by practitioners. This intrinsic principle is hidden behind the abundant optimization data and was not revealed by any previous studies, but it is easily explored by the enhanced gSOC-plus-BAB design. To further select between the two CVs, it is noted that the fans are located closer than the louvers to the manipulated variable of fan speed, then *c*_1_ = *P*_fan1_−*P*_fan2_ is finally chosen as the self-optimizing CV. Based on these ideal results, we do not need to further increase *n*_y_ for more complicated CVs.

**Fig. 9:**
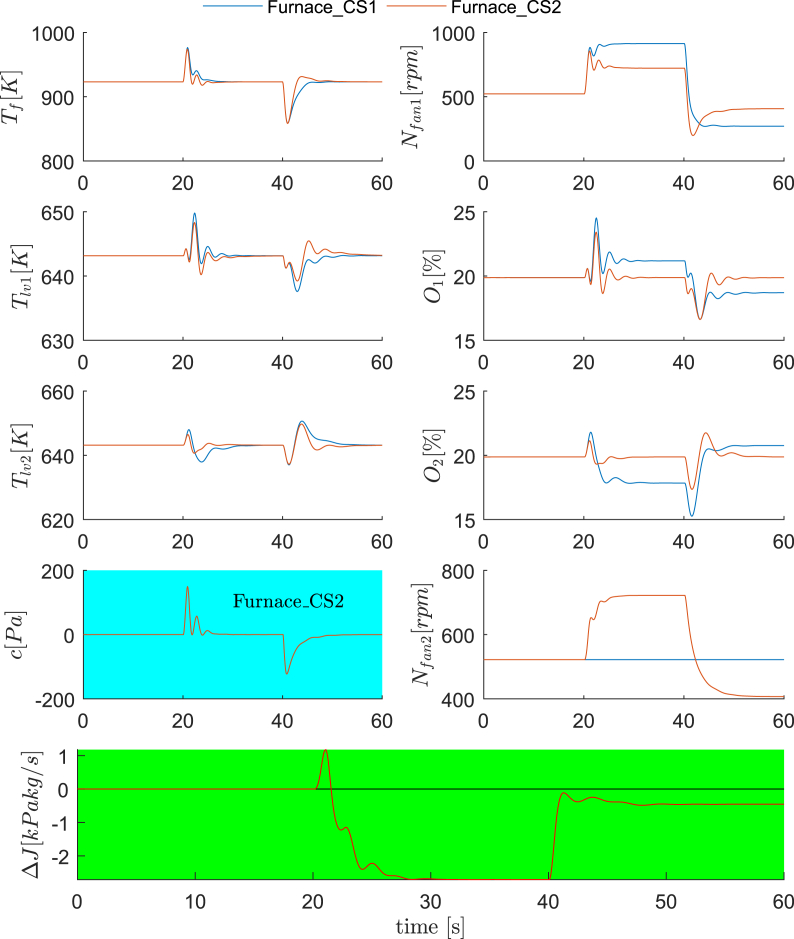
Dynamic comparisons between Furnace_CS1 and Furnace_CS2 (Scenario 1). 0–20 s: nominal condition; 20–40 s: D_F1_; 40–60 s: D_F2_.

**Fig. 10:**
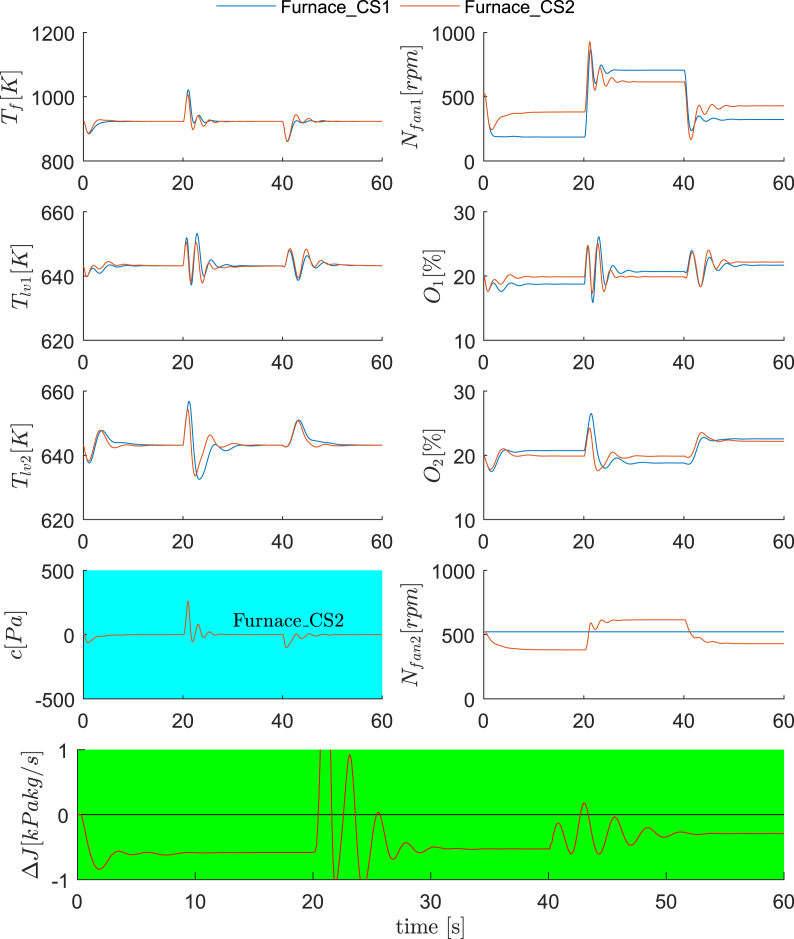
Dynamic comparisons between Furnace_CS1 and Furnace_CS2 (Scenario 2). 0–20 s: D_F5_; 20–40 s: D_F6_; 40–60 s: D_F9_.

**Dynamic validations**. The reconfigured control structure for the furnace subsystem is therefore the proposal of a new control loop *N*_fan2_↔*c*_1_, while keeping the other three the same as in Ref. [27], as shown in Fig. 8 (b). The control structure in Ref. [27] and the proposed new one are referred as Furnace_CS1 and Furnace_CS2, respectively. For dynamic validations, we arrange Scenario 3 and Scenario 4. Scenario 3 includes operating conditions for the nominal, D_F1_ and D_F2_ for every 20 s, while Scenario 4 includes D_F5_, D_F6_ and D_F7_ for the same durations. The dynamic responses of the control system and economic performances are illustrated in Fig. 9 and 10. One observes that the regulatory control qualities of Furnace_CS1 and Furnace_CS2 are again similar, some big oscillations occur for both control structures in Scenario 4, mostly attributing to the abrupt disturbances. On the other hand, the economic performance of Furnace_CS2 is superior than Furnace_CS1. Based on observed cost differences in the figures and Fig. 7, we confirm that the control structure Furnace_CS2 is indeed perfectly self-optimizing for the furnace.

Finally, these simulations support our claim that the plant economy benefits from the extremely simple control reconfiguration, without adding much complexity, if any. Such practice is deemed acceptable for industrial applications, in our view.

## 6 Conclusions

In this paper, we improved the Skogestad's systematical procedure for CSD of plant-wide chemical processes. Limitations of this state-of-art approach were discussed, mainly caused by the selection of single measurements as the self-optimizing CVs. The recent progress on the SOC methodology, particularly advanced approaches for selecting measurement combinations as CVs, open up the possibility of economic enhancements over the classical CSD procedure. Among various improved techniques, in this paper, we have recommended the gSOC-plus-BAB as an efficient toolkit for this purpose, which is particularly suitable for large-scale problems that cannot be easily addressed by other competing design approaches.

The enhanced CSD was applied to an industrial benchmarked off-gas system, whose control structures have been studied in-depth following the Skogestad's design procedure and turned out to be successful [27,33]. However, we were able to further improve their economic performances for both the roaster and furnace subsystems, by means of identifying measurement combinations as the self-optimizing CVs. Using the gSOC-plus-BAB method, promising self-optimizing CVs can be efficiently identified. In our study, the final recommended CVs are 0.494*P*_IDfan1_+0.506*P*_IDfan2_ (setpoint: −257.04) and *P*_fan1_−*P*_fan2_ (setpoint: 0) for the roaster and furnace subsystems, respectively. Both the two new recommendations require only mild modifications on the existing control structures [27,33]. In our view, these reconfiguration proposals are sufficiently simple, but their economic benefits are convincingly significant.

## Conflict of interest

The authors declare that they have no conflicts of interest to report regarding the present study.
